# Aspirin therapy in patients with acute respiratory distress syndrome (ARDS) is associated with reduced intensive care unit mortality: a prospective analysis

**DOI:** 10.1186/s13054-015-0846-4

**Published:** 2015-03-23

**Authors:** Andrew J Boyle, Stefania Di Gangi, Umar I Hamid, Linda-Jayne Mottram, Lia McNamee, Griania White, LJ Mark Cross, James J McNamee, Cecilia M O’Kane, Daniel F McAuley

**Affiliations:** Centre for Infection and Immunity, Health Sciences Building, Queen’s University Belfast, 97 Lisburn Road, Belfast, UK; Regional Intensive Care Unit, Royal Victoria Hospital, Grosvenor Road, Belfast, UK; Epidemiology ASL TO3, Via Sabaudia, 164, Grugliasco, TO 10095 Italy

## Abstract

**Introduction:**

Acute respiratory distress syndrome (ARDS) is a common clinical syndrome with high mortality and long-term morbidity. To date there is no effective pharmacological therapy. Aspirin therapy has recently been shown to reduce the risk of developing ARDS, but the effect of aspirin on established ARDS is unknown.

**Methods:**

In a single large regional medical and surgical ICU between December 2010 and July 2012, all patients with ARDS were prospectively identified and demographic, clinical, and laboratory variables were recorded retrospectively. Aspirin usage, both pre-hospital and during intensive care unit (ICU) stay, was included. The primary outcome was ICU mortality. We used univariate and multivariate logistic regression analyses to assess the impact of these variables on ICU mortality.

**Results:**

In total, 202 patients with ARDS were included; 56 (28%) of these received aspirin either pre-hospital, in the ICU, or both. Using multivariate logistic regression analysis, aspirin therapy, given either before or during hospital stay, was associated with a reduction in ICU mortality (odds ratio (OR) 0.38 (0.15 to 0.96) *P* = 0.04). Additional factors that predicted ICU mortality for patients with ARDS were vasopressor use (OR 2.09 (1.05 to 4.18) *P* = 0.04) and APACHE II score (OR 1.07 (1.02 to 1.13) *P* = 0.01). There was no effect upon ICU length of stay or hospital mortality.

**Conclusion:**

Aspirin therapy was associated with a reduced risk of ICU mortality. These data are the first to demonstrate a potential protective role for aspirin in patients with ARDS. Clinical trials to evaluate the role of aspirin as a pharmacological intervention for ARDS are needed.

## Introduction

Acute respiratory distress syndrome (ARDS) is a common devastating clinical syndrome characterised by life-threatening hypoxaemic respiratory failure often requiring mechanical ventilation and frequently leading to multiple organ failure. ARDS is a major cause of morbidity and mortality within the ICU, and causes long-term reduction in quality of life for survivors [1-3].

ARDS is an inflammatory condition characterised by neutrophil-mediated [4,5] and macrophage-mediated [6] injury. This uncontrolled local inflammatory response causes alveolar epithelial and capillary endothelial barrier damage, increasing its permeability. This allows the accumulation of an inflammatory infiltrate, and proteinaceous fluid within the alveolar space (non-cardiogenic pulmonary oedema) that contributes to profound hypoxaemia. The accompanying widespread activation of the coagulation cascade leads to microvascular thrombosis and fibroproliferation [7]. Currently there are few effective interventions for ARDS [8,9], and these primarily involve limiting ventilator-induced lung injury with low tidal volume ventilation [10], prone positioning [11], with emerging data for neuromuscular blockade [12] and extra-corporeal therapies [13,14].

Platelets have an increasingly recognised role in the inflammatory response leading to the development of ARDS. Platelet activation mediates neutrophil-recruitment to the lung in an acid-induced murine model of lung injury, an effect that is inhibited by pre-treatment with aspirin [15]. Platelet depletion in two mouse models of ARDS reduced the severity of lung injury and increased survival, an effect that was reproduced by pre-treatment with aspirin [16]. In addition, delayed neutrophil apoptosis is a feature of ARDS that aspirin can ameliorate to promote resolution of persisting inflammation [17].

Most, but not all [18,19] observational data suggest that aspirin use prior to ICU admission (without ARDS at the point of admission) may reduce the subsequent development of ARDS [20-22]. Finally, in a cohort of patients admitted with community-acquired pneumonia, those patients being treated with anti-platelet drugs (the majority of whom received aspirin) had a significantly lower rate of critical care admission [23].

These data suggest that aspirin may prevent ARDS, however, it is unknown if aspirin exposure alters outcome in patients with established ARDS. Aspirin exposure in patients with systemic inflammatory response syndrome (SIRS), severe sepsis or septic shock is associated with reduced mortality [24,25], but there are no data in patients with ARDS. We hypothesised that aspirin treatment, either prior to, or during ICU admission, would reduce mortality in patients with ARDS. To assess this, we prospectively identified patients with ARDS to determine the effect of aspirin exposure on mortality within ICU.

## Methods

We conducted an audit of ARDS management between December 2010 and July 2012. This was a convenience sample and a formal sample size calculation was not performed. The Belfast Health and Social Care Trust determined this project as an audit, because patient management was not altered, only routinely collected data were used and the data were fully anonymised, and as a result research ethics committee approval was not required. The requirement for patient consent was therefore waived.

### Patient population

All adult patients (>16 years-old) requiring invasive mechanical ventilation admitted to the 17-bed mixed medical and surgical Regional Intensive Care Unit in the Royal Victoria Hospital, Belfast, were prospectively screened daily as part of ongoing clinical trials of ARDS (53 of the patients included in this analysis were randomised to a clinical trial). Patients who met the North American-European consensus conference definition of acute lung injury [26] (a term now replaced by ARDS [27], and to which we refer throughout) were identified prospectively and included. There were no exclusion criteria. Determination of ARDS was made by trained research coordinators who are involved in the screening of patients with ARDS for recruitment into clinical trials ongoing within the ICU, and where there was any uncertainty the attending physician confirmed the diagnosis of ARDS. Patients were screened daily from admission for the diagnosis of ARDS, and were included in this cohort once they met the criteria for diagnosis.

### Data collection

Baseline demographics recorded were age, gender, and acute physiology and chronic health evaluation (APACHE) II score at admission to ICU. We recorded a medical history of alcohol dependency, smoking, liver cirrhosis, ischaemic heart disease, congestive heart failure, chronic obstructive airways disease, cerebrovascular disease and diabetes mellitus. We also recorded whether patients received a statin prior to admission. Medication history was obtained through chart review and medication reconciliation completed by the ICU pharmacist, who contacted the community general practitioner to complete these data if necessary. The ratio of partial pressure of arterial oxygen (PaO_2_) to fraction of inspired oxygen (FiO_2_), sequential organ failure assessment (SOFA) score on the day of ARDS diagnosis and the aetiology of ARDS was recorded. Aspirin usage both pre-admission and during ICU stay was included. Patients were deemed to have been treated with aspirin if it was part of their pre-hospital medication, or if given whilst in ICU (or both). All records were reviewed by a qualified physician.

### Primary and secondary outcomes

The primary outcome was ICU mortality. Secondary outcomes included duration of ICU stay and hospital mortality.

### Statistical methods

We compared patients with ARDS who received aspirin (aspirin ever) with those who did not (no aspirin). Baseline characteristics and outcome variables were compared using standard tests for continuous and binary variables. We reported results of the Kruskal-Wallis or chi squared test, as appropriate, when we compared three or more groups.

Data are presented as mean (SD), median (IQR) and number (%) as appropriate. The number of non-missing observations is shown when different from the overall population. The distributions of all variables were tested for normality. The independent sample Student’s *t-*test was used for continuous variables with a normal distribution. Otherwise, the Wilcoxon‐Mann‐Whitney was used when normality was violated. The chi squared test or Fisher’s exact two-sided test, as appropriate, were used for binary variables. A *P*-value ≤0.05 was considered statistically significant.

Univariate and multivariate logistic regression models were performed to identify the association between baseline characteristics and the primary outcome. We used multivariate logistic regressions to calculate (adjusted) odds ratios (OR) and 95% confidence intervals (CI) for the association between aspirin use and outcomes. Adjustments for any potential confounding effects were made for clinically relevant variables and for those that showed a statistically significant difference between the groups at baseline.

We further examined the adjusted effect of aspirin use on the primary and secondary outcomes with multivariate Cox proportional hazards regression models. The dates of admission to ICU or hospital, respectively, were the starting points of the survival analyses and the dates of discharge from ICU, or hospital, were the endpoints.

Multivariate models were constructed using automatic stepwise selection estimation with likelihood ratio testing (*P*-value ≤0.20) specified as the test of significance to include or exclude variables. Aspirin exposure (as the variable of interest), and history of coronary artery disease (as a significant, potential confounder) were locked into the model and not subject to the selection criteria. In a secondary analysis, combined aspirin and statin exposure, and history of coronary artery disease, were locked into the model. APACHE and SOFA as severity of illness scores were not both included in the multivariate model because of the problem of co-linearity in that the scores are correlated, because the severity of illness variables by which they are calculated are broadly the same or correlated. The Kaplan-Meier curve was used to plot ICU survival between patient groups with the log rank test used to determine differences among these groups. STATA SE version 11.0 (StataCorp LP, TX, USA) was used for all analyses. Graphs were constructed using GraphPad Prism (v 6.0).

## Results

Two hundred and two patients with ARDS were included; 56 (28%) received aspirin either pre-hospital (n = 31, 55% of total aspirin), in ICU (n = 7, 13%) or both pre-hospital and in ICU (n = 18, 32%). The dose of aspirin received ranged from 75 mg daily (n = 53, 95%) to 300 mg daily. Patient demographics are summarised in Table 1. Patients treated with aspirin were likely to be older (71 versus 56; *P* <0.001) and have greater co-morbidities. The use of statin therapy was higher in the aspirin group (37 versus 24; *P* = <0.001).

**Table 1:** **Baseline characteristics of patients with acute lung injury**

	**Overall**	**Aspirin ever**	**No aspirin**	***P*** **-value**
	**(n = 202)**	**(n = 56, 28%)**	**(n = 146, 72%)**	
Age				
Median	61	71	56	<0.001*
IQR	46 to 71	60 to 77	41 to 68	
Male				
Number (%)	129 (64)	40 (71)	89 (61)	0.17
APACHE II score				
Median	19	21	18	0.10
IQR	14 to 24	17 to 24	13 to 24	
SOFA				
Median	8	8	8	0.99
IQR	6 to 11	7 to 11	6 to 11	
PaO_2_/FiO_2_ ratio				
Median	22.9	21.1	23.5	0.08
IQR	17 to 30	16 to 27	18 to 31	
Vasopressor use				
Number (%)	95 (47)	30 (54)	65 (45)	0.25
Sepsis				
Number (%)	111 (55)	29 (52)	82 (56)	0.58
Coronary artery disease				
N (%)	33 (16)	22 (39)	11 (8)	**<0.001**
Cerebrovascular disease				
Number (%)	12 (6)	9 (16)	3 (2)	0.001*
COPD				
Number (%)	29 (14)	13 (23)	16 (11)	0.02*
Diabetes mellitus				
Number (%)	30 (15)	18 (32)	12 (8)	<0.001*
Smoker				
Number (%)	99 (49)	34 (61)	65 (45)	0.04*
Alcohol abuse/liver cirrhosis				
Number (%)	58 (29)	9 (16)	49 (34)	0.01*
Pre-admission statin				
Number (%)	61 (30)	37 (66)	24 (16)	<0.001*

*Values that were deemed significant. APACHE II, acute physiology and chronic health evaluation; SOFA, sequential organ failure assessment; PaO_2_, arterial oxygen partial pressure; FiO_2_, fraction of inspired oxygen; COPD, chronic obstructive pulmonary disease.

### ICU mortality

The primary analysis involved those who had taken aspirin ever compared with those never exposed. In univariate analysis four variables were associated with ICU mortality; patient age, APACHE II score, SOFA score and vasopressor use (Table 2). Although aspirin exposure was not associated with an impact upon mortality in the univariate analysis (Table 2), in a multiple logistic regression analysis, treatment with aspirin was associated with a significantly lower ICU mortality compared with patients who had no aspirin exposure (OR 0.38 (0.15 to 0.96); *P* = 0.04). The use of vasopressors during admission (OR 2.09 (1.05 to 4.18); *P* = 0.04) and APACHE II score at admission (OR 1.07 (1.02 to 1.13); *P* = 0.01) was associated with an increased risk of death (Table 3).

**Table 2:** **Univariate analysis of ICU mortality**

**Predictor**	**Univariate analysis**	
	**Odds ratio (95% CI)**	***P*** **-value**
Age	1.03 (1.00, 1.05)*	0.01*
Male	0.65 (0.34, 1.22)	0.18
APACHE II score	1.10 (1.05, 1.16)*	<0.001*
PaO_2_/FiO_2_ ratio	0.97 (0.93, 1.00)*	0.06*
SOFA score	1.35 (1.21, 1.50)*	<0.001*
Vasopressor use	2.54 (1.34, 4.81)*	0.004*
Aspirin use (ever)	0.75 (0.37, 1.53)*	0.43*
Smoking	0.82 (0.44, 1.53)	0.54
Coronary artery disease	1.20 (0.53, 2.71)*	0.67*
Diabetes mellitus	1.41 (0.61, 3.24)	0.42
Pre-admission statin	0.95 (0.46, 1.97)	0.90
COPD	0.83 (0.33, 2.07)	0.69
Cerebrovascular disease	0.88 (0.23, 3.40)	0.86
Alcohol abuse history or cirrhosis	1.30 (0.67, 2.54)	0.44
Sepsis	1.63 (0.86, 3.09)	0.13

*Values that were taken forward to multivariate analysis. APACHE II, acute physiology and chronic health evaluation; SOFA, sequential organ failure assessment; PaO_2_, arterial oxygen partial pressure; FiO_2_, fraction of inspired oxygen; COPD, chronic obstructive pulmonary disease.

**Table 3:** **Multivariate analysis of ICU mortality**

**Predictor**	**Odds ratio (95% CI)**	***P*** **-value**
Aspirin use	0.38 (0.15, 0.96)	0.04*
Age	1.02 (1.00, 1.05)	0.10
APACHE II score	1.07 (1.02, 1.13)	0.01*
Coronary artery disease	1.16 (0.43, 3.10)	0.77
PaO_2_/FiO_2_ ratio	0.97 (0.93, 1.00)	0.08
Vasopressor use	2.09 (1.05, 4.18)	0.04*

*Values that were deemed significant. APACHE II score on admission is presented as a continuous variable. APACHE II, acute physiology and chronic health evaluation; PaO_2_, arterial oxygen partial pressure; FiO_2_, fraction of inspired oxygen.

In analysis of the combined effect of aspirin and statin therapy, patients exposed to both drugs had no ICU survival advantage (OR 0.68 (0.26, 1.80); *P* = 0.44) (Table 4).

**Table 4:** **Multivariate model for analysis of ICU mortality including patients with combined aspirin and statin exposure**

**Predictor**	**Odds ratio (95% CI)**	***P*** **-value**
Aspirin and statin exposure	0.68 (0.26, 1.80)	0.44
Age	1.01 (0.99, 1.04)	0.21
APACHE II score	1.07 (1.02, 1.13)	0.01*
Coronary artery disease	1.01 (0.39, 2.63)	0.99
PaO_2_/FiO_2_ ratio	0.97 (0.94, 1.01)	0.15
Vasopressor use	2.06 (1.04, 4.08)	0.04*

*Values that were deemed significant. APACHE II score on admission is presented as a continuous variable.

**Figure 1:**
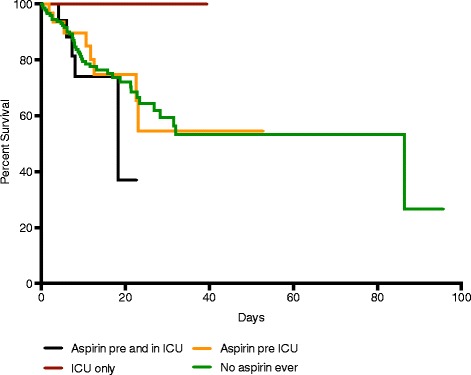
**ICU survival in different aspirin exposure groups.** Kaplan-Meier graph showing survival status for patients in the four different subgroups of aspirin treatment. There was no significant difference in survival amongst the groups (log rank test, *P* = 0.48).

To determine the effect of timing of treatment we analysed the different subgroups of patients treated with aspirin (aspirin pre-ICU, aspirin pre-ICU and in ICU, aspirin in ICU only) and those never treated with aspirin. Although there was increased survival in patients started on aspirin within ICU, there was no significant difference in survival amongst the groups (log rank test, *P* = 0.48) (Figure 1).

### Length of ICU stay

Aspirin treatment was not associated with a significant impact upon ICU length of stay (hazard ratio (HR) 0.64 (0.33 to 1.25), P = 0.19), whilst APACHE II score correlated with a longer ICU admission (HR 1.07 (1.03 to 1.12), P = 0.001) *P* = 0.001).

### Hospital mortality

The association of aspirin did not extend to overall hospital mortality (OR 0.91 (0.46 to 1.78); *P* = 0.78). APACHE II score positively correlated with hospital mortality (OR 1.09 (1.04 to 1.14); *P* <0.001).

## Discussion

In this prospectively identified audit of ARDS management, we found aspirin therapy to be associated with a reduced risk of ICU mortality from ARDS. Using multivariate analysis in patients with ARDS, we found aspirin was an independent factor associated with a reduction in risk of death within ICU, whilst the use of vasopressors and APACHE II score at ICU admission correlated with increased risk of ICU death. This is the first report to find aspirin therapy is associated with reduced ICU mortality in patients with established ARDS.

There are several processes that aspirin may modulate to provide a beneficial effect in ARDS. Aspirin has significant anti-platelet properties through the inhibition of cyclo-oxygenase enzymes that prevent thromboxane A2 production, therefore suppressing platelet aggregation; an effect that can last up to 10 days post administration [28]. Platelets aggregate at sites of lung injury and act as signalling molecules to propagate an immune response, facilitating the recruitment of neutrophils to the injured alveolus [15]. Our findings in this cohort could be explained by the anti-platelet effect of aspirin, which prevents neutrophil recruitment and infiltration of the alveolar space. This hypothesis is supported by animal models of ARDS that show reductions in neutrophil migration to the alveolar space in mice treated with aspirin [29], suggesting that aspirin therapy is effective in reducing the severity of ARDS by modulating platelet-neutrophil interactions.

Neutrophil recruitment to sites of lung injury may also be modulated through aspirin-triggered anti-inflammatory mediators. Aspirin acetylates cyclo-oxygenase-2, which results in the production of aspirin-triggered Resolvin D1 (AT-RvD1), an anti-inflammatory lipid mediator that can reduce neutrophil transendothelial migration in a peritonitis model [30]. In a murine model of ARDS, AT-RVD1 treatment was shown to reduce neutrophil and macrophage recruitment to the site of lung injury, in addition to restoring the capillary-endothelial alveolar-epithelial barrier function. This suggests that aspirin therapy can decrease severity and augment resolution of ARDS [31].

Delayed neutrophil apoptosis is a prominent feature of ARDS [32], allowing inflammatory cells to remain within the alveolar space, prolonging the period of lung injury and hypoxia. Aspirin has previously been shown to preserve neutrophil apoptosis [17] and in the setting of ARDS, experimental evidence suggests that aspirin-triggered production of the anti-inflammatory lipid mediator 15-epi-lipoxin A_4_ restores neutrophil apoptosis and enhances the resolution of alveolar inflammation [33]. The effect of aspirin may be dose-dependent [34] and although our data support the role of low-dose therapy, we require future clinical trials to investigate the most appropriate dose for aspirin in ARDS.

Prior observational studies involving aspirin and ARDS suggest an association between aspirin exposure and prevention of ARDS in at-risk groups [20-22], but as yet there is no evidence about aspirin use as a therapy for ARDS. In sepsis, the most common precipitant of ARDS, observational studies have shown reduced mortality when aspirin is administered during the course of illness [24,25]. In addition, there are clinical implications from our audit. In our cohort, 63% of pre-hospital aspirin users had their anti-platelet therapy discontinued at ICU admission, often without documented justification. Previous observational studies have shown reduced ICU mortality in patients exposed to aspirin prior to ICU admission [35], and the addition of our data suggests that consideration should be given to continuing aspirin at ICU admission provided no contraindications exist [36]. Pre-admission statin prescription was higher in the aspirin cohort. The combined effect of aspirin and statin treatment has previously been associated with a reduced incidence of ARDS [19], however in our cohort there was no association with an ICU survival advantage, suggesting that there is limited therapeutic benefit of combined treatment in ARDS. This finding adds to recent clinical trials suggesting that there is a limited role for statin therapy in ARDS [37,38].

Of the different subgroups of patients treated, those who were prescribed aspirin within ICU showed the greatest survival benefit although this did not reach statistical significance, which may have been related to the small patient number. We are cautious in our interpretation of this, and believe that further clinical trials are required to assess the benefit of aspirin as a treatment for ARDS.

Our patients were prospectively identified as part of ongoing screening for recruitment to clinical trials within our ICU, and we believe this identification process to be a strength that supports the reliability of our findings. We found APACHE II score and vasopressor use to be associated with increased ICU mortality from ARDS, and this finding confirms previous studies that have shown these as independent predictors of ICU mortality [39], supporting that this cohort is representative.

The limitations of our findings are in keeping those of observational studies. The aspirin cohort had higher rates of associated co-morbidity, and it could be that aspirin usage is a surrogate for an unknown confounding factor. We opted to combine patients who received aspirin pre-admission and during admission into a single cohort because of the prolonged inhibition of thromboxane A2 for up to 10 days [28]. However many of our patients did not receive aspirin within ICU. In addition, adverse events associated with the continuation of aspirin in ICU were not consistently recorded. Therefore, it is feasible that the efficacy and safety profile could be different when aspirin is used specifically as a treatment for ARDS in ICU. Although prior observational data suggest benefit in continuing aspirin in patients with a high risk of bleeding [35], it is important that the potential risks associated with aspirin in this patient population are clarified in future clinical trials of aspirin as a treatment for ARDS. These findings may have been confounded by the so-called healthy user effect [40], whereby the more unwell patients have their medications, including aspirin, discontinued. Although unable to control for this in our cohort, we believe the finding that both APACHE II and SOFA scores were similar between the groups suggests that this effect has not had a significant impact upon our findings. The use of ICU mortality as the primary outcome measure is a potential limitation, however we believe it reflects the outcome specifically from ARDS, whilst the cause of hospital mortality may be unrelated to the development of ARDS. However the lack of an association with aspirin usage and hospital mortality may indicate the association with aspirin and clinical outcomes is limited and therefore further research is required to confirm these findings. Finally, smoking and alcohol consumption histories were obtained from review of the medical chart. We acknowledge that this is a potential limitation in the assessment of these factors within our cohort and may not fully represent the confounding effects of smoking and alcohol [41].

At present our findings are hypothesis-generating, and support the need for clinical trials to investigate aspirin as a therapy for ARDS. In addition to trials investigating aspirin as a preventative treatment for ARDS [42], our group is currently investigating aspirin in a model of ARDS induced by inhaled lipopolysaccharide (LPS) in healthy volunteers (ARENA, NCT01659307) and are planning a phase-2 study of aspirin in patients with ARDS (STAR, NCT02326350). Both of these trials will better inform clinicians as to the clinical potential of aspirin as a therapy for ARDS.

## Conclusion

In summary, our audit demonstrates an association between aspirin use and reduced ICU mortality in patients with ARDS. Early-phase clinical trials investigating the potential for aspirin as a therapy for ARDS are needed.

## Key messages

- In this audit aspirin therapy is associated with reduced risk of ICU mortality in patients with ARDS.
- APACHE II score and vasopressor use correlate with ICU mortality.
- There is a need for clinical trials investigating the role for aspirin in ARDS.

## Abbreviations

APACHE: acute physiology and chronic health evaluation
ARDS: acute respiratory distress syndrome
AT-RvD1: aspirin-triggered Resolvin D1
FiO_2_: fraction of inspired oxygen
CI: Confidence Intervals
HR: hazard ratio
LPS: Lipopolysaccharide
OR: odds ratio
PaO_2_: partial pressure of oxygen
SIRS: systemic inflammatory response syndrome
SOFA: sequential organ failure assessment
